# Polymorphisms in the human serotonin receptor 1B (HTR1B) gene are associated with schizophrenia: a case control study

**DOI:** 10.1186/s12888-018-1849-x

**Published:** 2018-09-19

**Authors:** Xi Xia, Mei Ding, Jin-feng Xuan, Jia-xin Xing, Hao Pang, Bao-jie Wang, Jun Yao

**Affiliations:** School of Forensic Medicine, China Medical University, No. 77 Puhe Road, Shenbei New District, Shenyang, 110122 China

**Keywords:** Schizophrenia, Single-nucleotide polymorphisms (SNPs), Serotonin receptor 1B (HTR1B)

## Abstract

**Background:**

Schizophrenia is associated with multiple neurotransmitter disorders, including serotonin (5-hydroxytryptamine, 5-HT). The neuromodulatory action of serotonin on brain function largely depends on the action of specific subtypes of serotonin receptors. The serotonin receptor 1B (HTR1B) gene has been proposed to play putative roles in the development of multiple emotional and psychiatric disorders.

**Methods:**

To study the relationship of *HTR1B* polymorphisms and schizophrenia, gene information was drawn from a cohort of 310 schizophrenic patients (152 men and 158 women) and 313 healthy controls (153 men and 160 women) of northern Han Chinese descent. The χ2 test was used to compare allele and genotype distributions between case and control groups. The haplotype and linkage equilibrium were also assessed in two group comparisons.

**Results:**

We detected 14 SNPs. Male patients were observed to have higher frequencies of the A-allele and AA+AG genotype at rs1778258 than female patients (*p* = 0.012 and *p* = 0.015, respectively). Both the A-allele and AA+AG genotype were associated with schizophrenia risk (OR = 1.986 and OR = 2.061, respectively), although the statistical significance of the genotype was lost after Bonferroni correction. Linkage analysis showed that rs17273700, rs11568817, rs9361234 and rs58138557 polymorphisms exhibit strong linkage disequilibrium (LD). In addition, schizophrenic patients show stronger linkage between 11,568,817 and rs130058 than healthy controls.

**Conclusions:**

*HTR1B* polymorphisms are associated with schizophrenia in the northern Han Chinese population, which provides an etiological reference for schizophrenia.

## Background

Schizophrenia is a chronic disabling mental disorder affecting more than 21 million people worldwide, and is characterized by distortions in perception, thinking, emotions, sense of self and behavior. Common experiences include hearing voices and delusions (WHO2016). The etiology of schizophrenia is complicated, and genetic studies have had a guiding influence on schizophrenia research. A genome-wide association study (GWAS) suggested that schizophrenia is a complex polygenetic disease with over 80% heritability [1]. Numerous studies have focused on the neurotransmitters related to the pathogenesis of schizophrenia, including serotonin. Serotonin plays an import role in various brain activities including emotions, pain, assault, learning and memory [2].

The neuromodulatory action of serotonin on brain function largely depends on the actions of serotonin receptors, which comprise at least 14 different classes of subtypes [3]. HTR1B is a G-protein coupled receptor that activates a second messenger cascade to mediate inhibitory neurotransmission and regulate the release of serotonin, dopamine, and acetylcholine in the brain. HTR1B has been suggested to be associated with multiple emotional and psychiatric problems, including attention deficit hyperactivity disorder (ADHD) [4], antisocial behavior [5], aggressive behavior [6], bipolar disorder, anxiety/depression, schizophrenia [7, 8] and substance abuse [9]. It is also a common target for psychotherapeutic drugs. The *HTR1B* gene is located on chromosome 6 at position 77,460,848–77,464,022 (GRCh38.p7). It has been suggested that schizophrenic patients show increased *HTR1B* mRNA levels in the hippocampus. Simultaneous upregulation of *HTR1B* and downregulation of *HTR2A* could decrease GABAergic activity, which leads to an increased glutamatergic efferent in the hippocampus [7]. In previous studies, there were inconsistent results on the genetic association between *HTR1B* gene variations and schizophrenia. For example, rs2143823 is considered to be related to schizophrenia in Croatians [10]. However, there was no correlation between G861C and schizophrenia in Portuguese, German and Brazilian patients [11–13]. The associations between C129T, T371G, T655C, C705T, G861C, A1099G, G1120A and schizophrenia were also negative in mixed populations in the United States [14]. In addition, the *HTR1B* haplotype may be implicated in the gender discrepancy of schizophrenia in Spanish populations [15].

As the function of the coding regions has been studied extensively, SNPs located in regulatory regions were taken in to consideration. Prior studies addressing the association between schizophrenia and *HTR1B* polymorphisms in the Chinese Han population include only the 5′-untranslated (5’UTR) and coding regions, with little data concerning the 5′-promoter and 3′-regulatory regions. Therefore, we chose to investigate these regions to improve etiologic knowledge of this disease. Expanding sequences were investigated in our study, including a 2285 bp 5′-promoter region and a 1277 bp 3′-untranslated region, in the northern Han Chinese population to further explore the relationship between *HTR1B* and schizophrenia.

## Materials

### Subjects

Our sample comprised 310 schizophrenic patients (152 men and 158 women) and 313 genetically unrelated, healthy volunteers (153 men and 160 women), comprising a combined 623 individuals. All participants enrolled were of northern Han Chinese descent. Blood samples from schizophrenic patients were provided by the Third People’s Hospital of Liaoning Province; those from healthy controls were supplied by the China Medical University’s Forensic Evidence Department. Inclusion criteria included a diagnosis of schizophrenia by trained psychiatrists according to the Diagnostic and Statistical Manual of Mental Disorders, fourth edition (DSM-IV). Exclusion criteria included the presence of other psychiatric disorders. The results of patient questionnaires confirming no history nor present evidence of any psychiatric disorder and no history of mental disease for at least three prior generations were used to select healthy controls. All subjects provided written informed consent prior to enrollment in this study.

### SNP selection

The following factors were considered for SNP selection: (1) as the function of the *HTR1B* coding region has been studied extensively, SNPs located within *HTR1B* regulatory regions were selected; (2) based on prior reports, we found that current knowledge of the association between schizophrenia and *HTR1B* polymorphisms in the Chinese Han population include the 5′ untranslated region (UTR), while the promoter and 3′ regulatory regions have not been well studied; and (3) according to the *HTR1B* polymorphism distribution, we selected SNPs with favorable polymorphisms (MAF ≥ 0.1). Considering that the regulatory effects of sequences that are distal to the coding region may be limited, we selected a 2285 bp 5′-promoter region and a 1277 bp 3′-untranslated region for SNP investigation.

### DNA isolation and genotyping

Genomic DNA was extracted by the phenol-chloroform method from whole blood [16]. A 2285 bp fragment in the 5′-promoter region and a 1277 bp fragment in the 3′-untranslated region were included in our analysis. Standard PCR was performed in a total reaction volume of 20 μl (adjusted with sterilized, deionized water) containing 2 μl genomic DNA as template (approximately 50 ng), 10 μl of 2 × GC Buffer, 0.2 μl (1 U) of Taq polymerase (Takara LA Taq, Dalian, China), 2 μl (3.75 nmol) of dNTPs, 1.5 μl (7.5 pmol) each of sense and antisense primers. The primer sequences of the 5′-end fragment were 5’-TGGGTTTGTGCTTTATTGCCTT-3′ (sense), 5’-GGAGCAGAGGATAAGTTGGCTTG-3′ (antisense), and the primer sequences of the 3′-end fragment were 5’-CCCTTCTTCATCATCTCCCTAGTG-3′ (sense), 5’-ACCCCATTCCTCAATTGTGTAAG-3′ (antisense; Taihe Biotechnology Co., Beijing, China). PCR conditions for the 5′-end fragment were: 94 °C for 1 min; 35 cycles of 94 °C for 30 s, 58.4 °C for 30 s, and 72 °C for 1.5 min; then final extension at 72 °C for 7 min. PCR conditions for the 3′-end fragment were 94 °C for 1 min; by 35 cycles of 94 °C for 30 s, 60 °C for 30 s, 72 °C for 1 min; and extension at 72°Cfor 7 min. The Sanger double-chain termination method (Taihe Biotechnology Co., Beijing, China) was employed for DNA sequencing.

### Statistical analysis

Allele and genotype frequencies were calculated by direct counting. Haplotype blocks were determined by the confidence interval method in Haploview. Using this method, six 5′-promoter region SNPs were included in a haplotype block, while the eight 3′-promoter region SNPs were not included in a haplotype block. Thus, we performed analysis for haplotypes formed by the 6 SNPs in the block (namely, the 5′ block) and the remaining eight SNPs (designated the 3′ block) [17].

In order to test Hardy-Weinberg equilibrium (HWE) and construct haplotype blocks, linkage equilibrium (LD) analysis (D’ and r^2^) were performed using Haploview version 4.2 software (Broad Institute, Cambridge, MA, USA) [18]. The *χ*^*2*^ test was used to estimate correlations between the variance of polymorphism frequency distribution and schizophrenia. Statistical significance was defined as *p* < 0.05 (two-tailed). Statistical analyses were performed using SPSS Software19.0 (IBM, Armonk, NY, USA). A Bonferroni correction was applied for multiple comparisons to control for type I error, and the *p*-value was divided by the total number of loci or haplotypes [17].

## Results

We identified 14 SNPs in the present study (Table 1). The genotype distribution was in accordance with Hardy-Weinberg equilibrium in the control group. A summary of allele and genotype frequencies is presented in Table 2. We found that male patients were observed to have a significantly higher A-allele frequency at rs1778258 than female patients (*p* = 0.012). The frequency of A-allele carriers (AA+AG genotype) among male patients was also significantly higher than among female patients (*p* = 0.015), although statistical significance was lost after Bonferroni correction. The presence of both the A-allele and AA+AG genotype increased schizophrenia risk (OR = 1.986 and OR = 2.061, respectively; Table 3). We found no significant associations between any other SNPs and schizophrenia.

**Table 1:** SNPs (14 total) detected in the northern Han Chinese population

SNP	Chr. pos.	Base change^1,2^	MAF	REGION
rs4140535	77,465,335	− 1932 T > C	0.375	5’near gene
rs1778258	77,464,492	− 1089 G > A	0.113	5’near gene
rs17273700	77,464,263	− 860 T > C	0.121	5’near gene
rs1228814	77,464,103	−700 C > A	0.166	5’near gene
rs11568817	77,463,665	− 262 T > G	0.116	5’UTR
rs130058	77,463,564	−161 A > T	0.077	5’UTR
rs6297	77,462,224	*7 A > G	0.112	3’UTR
rs3827804	77,462,215	*16 G > A	0.011	3’UTR
rs140792648	77,462,065	*165_*166 Ins AG	0.029	3’UTR
rs9361234	77,461,965	*266 C > T	0.113	3’UTR
rs183156887	77,461,962	*269 C > A	0.0064	3’UTR
rs76194807	77,461,898	*333 G > T	0.113	3’UTR
rs58138557	77,461,771	*459_*460 Del GG	0.115	3’UTR
rs13212041	77,461,407	*824 A > G	0.256	3’UTR

^1^The A of the ATG start codon is designated as position 0

^2^An asterisk (*) indicates alterations in the coding region, which is 1173 bp in length

**Table 2:** Allelic distributions of the 14 SNPs and their associations with schizophrenia

			Case (*n*=310)		Control (*n*=313)		*p* Value^2^						
SNP	Allele^1^	Genotype	No.	Frequency	No.	Frequency	Allele^1^	Codominant (AA/aa,Aa/aa,AA/Aa)^3^			Recessive	Dominant	Overdominant
rs4140535							0.725	0.806	1.000	0.729	0.909	0.684	0.764
	T		394	0.635	391	0.625							
		CC	44	0.142	46	0.147							
		CT	138	0.445	143	0.457							
		TT	128	0.413	124	0.396							
rs1778258							0.585	1.000	1.000	0.541	1.000	0.552	0.542
	G		556	0.897	555	0.887							
		AA	4	0.013	4	0.013							
		AG	56	0.181	63	0.201							
		GG	250	0.806	246	0.786							
rs17273700							0.423	0.772	0.377	0.185	0.576	0.279	0.184
	T		554	0.894	550	0.879							
		CC	7	0.023	5	0.016							
		CT	52	0.168	66	0.211							
		TT	251	0.810	242	0.773							
rs1228814							1.000	0.374	0.247	0.465	0.276	0.663	0.356
	C		517	0.834	522	0.834							
		AA	13	0.042	8	0.026							
		AC	77	0.248	88	0.281							
		CC	220	0.710	217	0.693							
rs11568817							0.315	1.000	1.000	0.254	1.000	0.271	0.255
	T		559	0.902	553	0.883							
		GG	5	0.016	5	0.016							
		GT	51	0.165	63	0.201							
		TT	254	0.819	245	0.783							
rs130058							0.916	0.686	0.816	0.434	1.000	0.449	0.816
	T		49	0.079	48	0.077							
		AA	265	0.855	267	0.853							
		AT	41	0.132	44	0.141							
		TT	4	0.013	2	0.006							
rs6297							0.409	0.599	0.786	0.565	0.788	0.460	0.816
	A		541	0.873	536	0.856							
		AA	237	0.765	231	0.738							
		AG	67	0.216	74	0.236							
		GG	6	0.019	8	0.026							
rs3827804							0.802	—	0.800	—	0.800	—	0.800
	A		8	0.013	7	0.011							
		AG	8	0.026	7	0.022							
		GG	302	0.974	306	0.978							
		AA	0	0.000	0	0.000							
rs140792648							0.862	—	—	0.860	—	0.860	0.860
	—		604	0.974	608	0.971							
		—/—	294	0.948	295	0.942							
		—/AG	16	0.052	18	0.058							
		AG/AG	0	0.000	0	0.000							
rs9361234							0.408	1.000	1.000	0.348	1.000	0.364	0.349
	C		559	0.902	555	0.887							
		CC	254	0.819	247	0.789							
		CT	51	0.165	61	0.195							
		TT	5	0.016	5	0.016							
rs183156887							1.000	—	1.000	—	1.000	—	1.000
	A		4	0.006	4	0.006							
		AC	4	0.013	4	0.013							
		CC	306	0.987	309	0.987							
		AA	0	0.000	0	0.000							
rs76194807							0.486	0.725	0.242	0.472	0.340	0.725	0.242
	T		79	0.127	71	0.113							
		GG	234	0.755	247	0.789							
		GT	73	0.235	61	0.195							
		TT	3	0.010	5	0.016							
rs58138557							0.411	1.000	1.000	0.351	1.000	0.367	0.352
	GG		558	0.900	554	0.885							
		—/—	5	0.016	5	0.016							
		—/GG	52	0.168	62	0.198							
		GG/GG	253	0.816	246	0.786							
rs13212041							0.948	0.643	0.444	0.423	0.629	0.545	0.361
	G		160	0.258	160	0.256							
		AA	171	0.552	179	0.572							
		AG	118	0.381	108	0.345							
		GG	21	0.068	26	0.083							

^1^The allele for each SNP indicated is that with the higher patient frequency.

^2^The significance levels of chi-squared tests for codominant, dominant, recessive and overdominant model were adjusted using the Bonferroni correction (significance level *p* = 0.0036).

^3^The SNP allele that is observed at a higher frequency in patients is designated A; the other allele is designated a.

**Table 3:** The relationship between rs1778258 and schizophrenia according to biological sex

Group	rs1778258	Male	Female	*p* value^1^	OR	95% CI
case	A	41/304	23/316	0.012	1.986	1.161–3.398
	AA+AG	38/152	22/158	0.015	2.061	1.152–3.684
control	A	33/306	38/320	0.706	0.897	0.547–1.472
	AA+AG	38/153	22/160	0.891	0.945	0.550–1.622

^1^A significance level *p* = 0.05

**Fig. 1:**
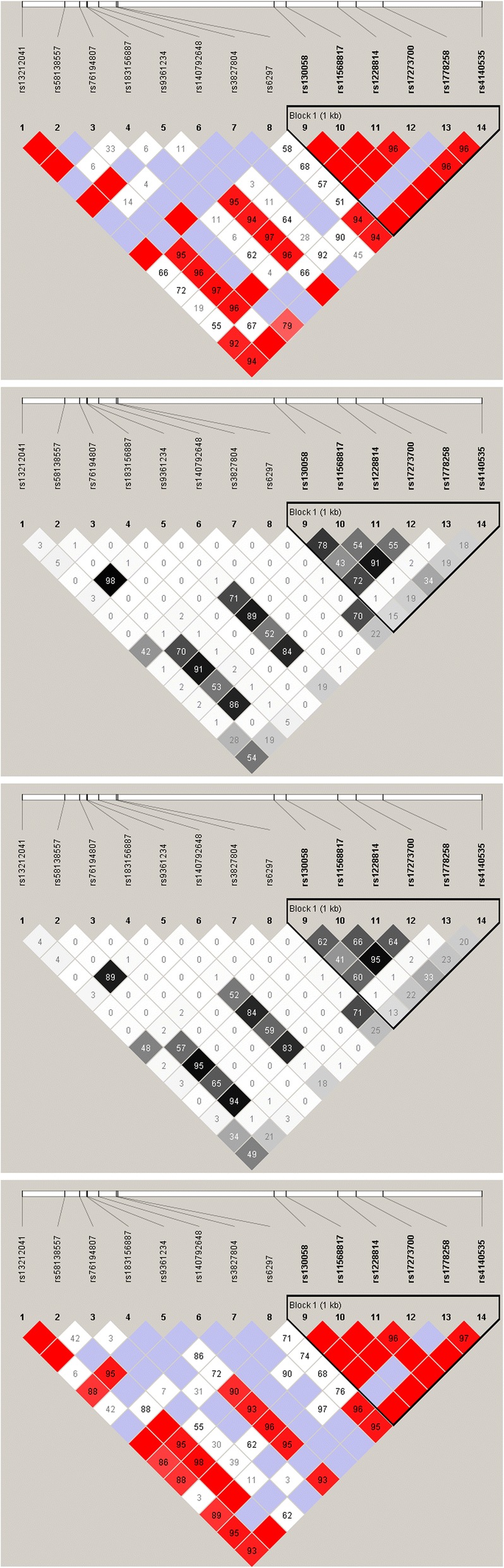
Linkage disequilibrium diagram of the 14 SNPs in HTR1B in the northern Han Chinese population. Cases D’. Cases r^2^. Controls D’. Controls r^2^. The number in the grid of the red-white diagram represents the D’ value, the deep red grid represents D’ 1, and the number in the grid of the black-white diagram represents the r^2^ value

Via linkage disequilibrium (LD) analysis, rs11568817 was found to be in strong linkage disequilibrium with rs17273700, rs9361234 and rs58138557 in northern Han Chinese patients (r^2^ > 0.8) [18]. We also found that, as compared to the healthy people, the linkage between rs11568817 and rs130058 in schizophrenic patients was more intense. Furthermore, LD analysis showed that the six 5′-promoter region SNPs are included in a haplotype using the confidence interval method, while the eight 3′-promoter region SNPs were not included in a haplotype block (Fig. 1). We next compared the frequencies of haplotypes formed by the six SNPs in block or the remaining eight SNPs between the patients and healthy people, and found that the haplotype frequency distributions for the 5′-promoter region C-G-C-A-G-A were significantly different among individuals in the case and control groups. (*p* = 0.044); however, statistical significance was lost after Bonferroni correction. No statistical differences between groups were observed for other haplotypes (Tables 4 and 5).

**Table 4:** The relationship between 5′-region haplotype and schizophrenia

Haplotype							Case(*n* = 310)		Control (*n* = 313)				
5’	rs4140535	rs1778258	rs17273700	rs1228814	rs11568817	rs130058	N	F	N	F	*p* value^1^	OR	95% CI
1	T	G	T	C	T	A	391.840	0.632	389.998	0.623	0.770	1.040	0.827–1.309
2	C	A	T	C	T	A	62.620	0.101	70.112	0.112	0.583	0.898	0.627–1.288
3	C	G	T	C	T	A	58.900	0.095	58.844	0.094	1.000	0.011	0.692–1.477
4	C	G	C	A	G	T	48.980	0.079	48.202	0.077	0.916	1.033	0.683–1.564
5	C	G	T	A	T	A	39.060	0.063	30.048	0.048	0.266	1.334	0.817–2.176
6	C	G	C	A	G	A	11.780	0.019	25.040	0.040	0.044	0.474	0.236–0.953

^1^Significance level *p* = 0.01

**Table 5:** The relationship between 3′-region haplotype and schizophrenia

Haplotype									Case (*n* = 310)		Control (*n* = 313)				
3’	rs6297	rs3827804	rs140792648	rs9361234	rs183156887	rs76194807	rs58138557	rs13212041	N	frequency	N	frequency	*p* value^1^	OR	95% CI
1	A	G	A	C	C	G	T	A	298.220	0.481	295.472	0.472	0.777	1.038	0.831–1.297
2	A	G	A	C	C	G	T	G	78.740	0.127	68.234	0.109	0.334	1.198	0.849–1.692
3	G	G	A	C	C	G	T	G	78.120	0.126	88.892	0.142	0.407	0.868	0.626–1.204
4	A	G	A	C	C	T	T	A	76.260	0.123	68.860	0.110	0.537	1.128	0.797–1.595
5	A	G	A	T	C	G	A	A	58.900	0.095	68.234	0.109	0.455	0.863	0.597–1.247
6	A	G	T	C	C	G	T	A	16.120	0.026	16.276	0.026	1.000	1.010	0.501–2.038
7	A	A	A	C	C	G	T	A	8.060	0.013	6.886	0.011	0.802	1.156	0.417–3.207

^1^Significance level = 0.0083

## Discussion

In the present study, we investigated *HTR1B* polymorphisms in 623 individuals of northern Han Chinese descent, including 310 schizophrenic patients and 313 healthy controls. We ultimately detected 14 SNPs. According to previously reported observations, no evidence regarding a relationship between *HTR1B* and schizophrenia was found in any allele or major haplotype for T-261G, -182INS/DEL-181, A-161 T, C129T and G861C in Han Chinese patients [19]. Our study showed consistent results regarding rs11568817 (T-261G) and rs130058 (A-161 T). When patients were grouped by gender, male patients were observed to have a significantly higher frequency of A-allele at rs1778258 than female patients (*p* = 0.012). Based on multiple comparisons of genotype effects, we inferred that the most probable mode of inheritance of rs1778258 is the dominant model [20]. The frequency of the AA+AG genotype in male patients was also significantly higher than in female patients (*p* = 0.015), although statistical significance was lost after Bonferroni correction. Both the A-allele and AA+AG genotype were associated with increased risk of schizophrenia (OR = 1.986 and OR = 2.061, respectively). Thus, our study provides evidence that rs1778258 involved with gender variations in schizophrenia.

It is understood that sex differences exist in brain function as well as the vulnerability, incidence, manifestation, and treatment of numerous psychiatric diseases, which are determined by inherent biological differences between males and females. For instance, males show a higher propensity for Parkinson’s disease, autism, attention deficit hyperactivity disorder (ADHD) and addiction. Females also show higher susceptibility to Alzheimer’s disease and anxiety/depression [21]. Most studies have found the onset age of schizophrenia to be earlier in men than in women, although there is no sex difference in overall incidence of schizophrenia [22]. Men with schizophrenia also present with more cognitive disturbances and greater reductions in temporal lobe volume than women with schizophrenia [23]. Moreover, evidence also supports sex differences in serotonin neurotransmission and psychiatric disorders caused by malfunctions in the serotonin system. Such differences are not only due to hormonal regulation, but are also attributable genetic effects [21, 24]. It was previously reported that higher whole blood 5-HT levels in women than in men are influenced by multiple genes on chromosome 2, 6, and 17 [25]. In addition, the effects of sex hormones on serotonin regulation have also been reported. It has been shown that estradiol plays a protective role against the cognitive, positive, and negative symptom domains of schizophrenia [26]. Our results indicated that rs1778258 is associated with gender in schizophrenia. Since rs1778258 is located in the promoter region, it may have an influence on gene expression. Alternatively, there could be a functional site closely linked to rs1778258, and its variation thus could modify gene expression [27], which could have an influence on the serotonin system.

Previous genetic studies on schizophrenia have suggested that there is a gender-specific association between select dopamine genes and schizophrenia. Gender-specific associations between genotype and schizophrenia were also observed in GABAergic-mediated regulation of anterior cingulate cortex function. Male schizophrenic patients expressed significantly lower levels of *GABA-Aα5*, *GABA-Aβ1*, *and GABA-Aε*, while the expression of *GABA-Aβ1* and *GAD67* was significantly higher in female patients, as compared to sex-matched controls [28]. Since it is known that these neurotransmitter systems are closely related [29], these differences may contribute to the gender-specific relationship between *HTR1B* polymorphisms and schizophrenia. Although sex differences are essential to study to fully understand the etiology of schizophrenia, unfortunately, few studies have been made regarding gender-specific associations between *HTR1B* and schizophrenia. The only association found to date was described in a Spanish population. The AAAC haplotype comprising rs6297, rs130058, rs1213366, and rs1213371 is significantly more frequent in female Spanish patients [15], which corroborates our finding that *HTR1B* genotype is related to gender in schizophrenia. It should be noted that rs1778258 was found to have a medium linkage with rs6297 (r^2^ = 0.7) in our study. However, both rs1778258 and rs6297 were found to lack any direct functional effects. Other SNPs had not been found to be associated with schizophrenia, but still provide references for *HTR1B* gene polymorphisms in the northern Han Chinese population.

This study found that the frequency distributions of haplotype C-G-C-A-G-A in the 5′-promoter region are significantly different between patients and healthy controls (*p* = 0.044), but statistical significance was lost after Bonferroni correction. It has been proposed that the rs11568817 G-allele and rs130058 T-allele influence gene expression by modifying the binding of transcription factors (TFs). The rs11568817 G-allele does enhance transcriptional activity; conversely, rs130058 T-allele can reverse this [30]. In our study, only haplotypes C-G-C-A-G-T and C-G-C-A-G-A contained functional sites rs11568817 G-allele or rs130058 T-allele, but no significant differences were found between patients and healthy controls in either haplotype after Bonferroni correction (*p* = 0.916 and *p* = 0.044, respectively. We also found that the 3′ region contains a known functional SNP, rs13212041. KP Jensen et al. confirmed that the rs13212041 A-allele can combine with microRNA (miR-96) to induce mRNA repression, and that this microRNA-mediated mRNA silencing could be attenuated by rs13212041 G-allele [31]. Conner et al. found that men with low expression haplotypes are inclined to present greater anger and hostility [27]. Although three known functional sites (rs11568817, rs130058, rs13212041) were found in our study, we discovered no direct associations between *HTR1B* and schizophrenia by haplotype. We hypothesize that the onset of schizophrenia could be affected by multiple functional sites, but their effects are counteracted [30]. Moreover, schizophrenia is associated with multiple neurotransmitters that have been noted to have physical and functional interactions [32]. Even these sites lead to changes in the serotonin system, but do not necessarily cause schizophrenia.

Our study showed that rs9361234, rs11568817 and rs17273700 are in strong linkage disequilibrium in the northern Han Chinese population. The linkage states of these SNPs are similar to those identified in Han Chinese populations in Beijing and southern China (1000 Genomes Project). We observed that rs58138557 was is intensely linked to rs9361234, rs11568817 and rs17273700 (Fig. 1). In addition, schizophrenic patients show stronger linkage between 11,568,817 and rs130058 than healthy controls (r^2^ = 0.78 and r^2^ = 0.62, respectively; Fig. 1). Linkage disequilibrium is related to population. For example, the linkage between rs11568817 and rs130058 in Han Chinese is weaker in northern China than in southern China based on the 1000 Genomes Project. This difference is also found in European and African-American populations [30]. Moreover, schizophrenia-associated genes vary among distinct ethnic populations [33]. Therefore, linkage disequilibrium of *HTR1B* could be associated with schizophrenia in northern Han Chinese peoples. This study presents information on the linkage state of *HTR1B* in schizophrenic patients of northern Han Chinese descent, and thereby provided references for the etiology of schizophrenia in different populations.

This study is limited by sample size and methods and, although we found that rs1778258 is related to gender in schizophrenia, unfortunately, the mechanism underlying this association was not addressed. This will require further exploration in the future. Because of the limitation of the candidate gene approach, caution must be taken in regards to the interpretation of the association we have observed. Since schizophrenia is a complex disease that is influenced by both genetic and environmental factors, both biological and psychological events experienced by individuals could have an impact on its onset. Thus, any associations cannot be fully explained by simply reducing them to a two-dimensional relationship between genetic variance and disease. We suggest that genetic background and behavioral events of enrolled patients should be taken into consideration [34]. Furthermore we will attempt to implement analysis of accurately defined phenotypes involving scale scores and treatment response, as well as the convergent analysis of genetic, serum and brain imaging markers.

## Conclusion

In this study, the *HTR1B* gene was found to be related to gender in schizophrenia, as the rs1778258 A-allele caused increased risk of schizophrenia in male patients. Linkage between rs11568817 and rs130058 is more intense in schizophrenic patients. Combining all results of this study, we assert that *HTR1B* has a putative relationship with schizophrenia in the northern Han Chinese population, which provides a reference schizophrenia etiology.

## Abbreviations

5-HT: 5-hydroxytryptamine
ADHD: Attention deficit hyperactivity disorder
DSM: Diagnostic Criteria of American Diagnostic and Statistical Manual of Mental Disorders
GWAS: Genome-wide association study
HTR1B: Serotonin receptor 1B
LD: Linkage disequilibrium
SNP: Single-nucleotide polymorphism
UTR: Untranslated region
